# Modest protection from vaccination against influenza A(H3N2) subclade K, Beijing, China, 2025/26 season

**DOI:** 10.2807/1560-7917.ES.2026.31.7.2600096

**Published:** 2026-02-19

**Authors:** Ying Shen, Daitao Zhang, Zhaomin Feng, Chunna Ma, Weixian Shi, Wei Duan, Jia Li, Lu Zhang, Dan Wu, Jiaojiao Zhang, Jiaxin Ma, Yingying Wang, Xiaodi Hu, Shuning Yan, Yuanzhi Di, Jiachen Zhao, Hui Xu, Guilan Lu, Yimeng Liu, Weijia Zhang, Quanyi Wang, Peng Yang

**Affiliations:** 1 Beijing Key Laboratory of Surveillance, Early Warning and Pathogen Research on Emerging Infectious Diseases, Beijing Center for Disease Prevention and Control, Beijing, China; 2 Beijing Research Center for Respiratory Infectious Diseases, Beijing, China; 3 School of Public Health, Capital Medical University, Beijing, China; * These authors contributed equally to this work and share first authorship.; ** These authors contributed equally to this work and share last authorship.

**Keywords:** vaccine effectiveness, influenza, A(H3N2), subclade K, China

## Abstract

During the early 2025/26 influenza season, influenza A(H3N2) subclade K rapidly predominated in Beijing, China. Using a test-negative design, we estimated influenza vaccine effectiveness (VE) among influenza-like illness outpatients tested between weeks 40/2025 and 04/2026. Among 10,484 participants, sequencing of 316 randomly selected A(H3N2)-positive samples showed 84.8% were subclade K, and antigenic analysis of 65 viruses indicated antigenic divergence. Despite this, adjusted VE against laboratory-confirmed influenza was 23.5% (95% confidence interval: 11.7–33.7), indicating modest protection during this subclade K-dominated season.

The 2025/26 influenza season in the northern hemisphere has been characterised by the early and rapid expansion of influenza A(H3N2) subclade K, with increased activity in multiple countries [1-3]. This subclade carries multiple amino acid substitutions in the haemagglutinin protein compared with the current influenza vaccine strain, raising concerns about partial immune escape and reduced vaccine effectiveness (VE) [4,5]. Although early real-world VE estimates against laboratory-confirmed influenza during subclade K-dominated seasons have been reported, most evidence to date comes from European surveillance networks, and data from other regions remain limited [6-8]. In Beijing, influenza activity started in September, earlier than in most previous seasons, peaked in early December, and subsequently declined. Using data from the Beijing influenza surveillance system, we provide interim estimates of 2025/26 seasonal influenza VE during a period of predominant subclade K circulation. For context, influenza vaccination coverage in Beijing was highest among school-aged children (approximately 58%) and adults aged ≥ 60 years (approximately 18%) in 2025/26 season.

## Study design and data sources

We conducted a test-negative design study on these surveillance data, covering 40 sentinel hospitals and 19 laboratories in Beijing, China [9]. The study population consisted of outpatients of all ages presenting with influenza-like illness (ILI), defined as axillary temperature ≥ 38 °C with cough or sore throat, with symptom onset within 3 days before sampling between weeks 40/2025 (approximately 2 weeks after the start of the mass vaccination campaign) and 04/2026. In China, individuals with ILI symptoms can access hospital outpatient services directly. Therefore, the majority of outpatient ILI cases are mild to moderate in severity. We append an overview of the enrolment over time in Supplementary Figure S1 [8]. Oropharyngeal swabs were collected and tested by real-time reverse transcription PCR (RT-PCR). To ensure balanced ILI selection, we recruited approximately five patients daily per sentinel hospital throughout the year. A subset of positive specimens underwent whole genome sequencing and antigenic characterisation using hemagglutination inhibition (HI) assays.

Cases were ILI patients with PCR-confirmed influenza, and controls were ILI patients testing negative. We verified vaccination status using the immunisation planning information management system of the Beijing Center for Disease Prevention and Control (CDC). In the primary analysis, participants were considered vaccinated in the current season if the vaccine was administered ≥ 14 days before symptom onset; those vaccinated 0–14 days before symptom onset were excluded. We defined prior-season vaccination as receipt of ≥ 1 dose of seasonal influenza vaccine in the 2024/25 season.

Vaccine effectiveness (VE) was estimated as (1 − OR) × 100% using a logistic regression model adjusting for age (modelled as a natural cubic spline), sex, presence of at least one comorbidity, area of residence, and calendar week (modelled as a natural cubic spline) [10,11]. We also conducted age-stratified analyses. We performed sensitivity analyses to assess the impact of different vaccination definitions and enrolment periods on VE estimates.

## Characteristics of the study population

In the primary analysis, between weeks 40/2025 and 04/2026, we recruited a total of 13,130 ILI outpatients. We excluded 2,646 participants, including eight with missing information on influenza subtype, 2,497 without documented vaccination records and 141 vaccinated 0–14 days before symptom onset. People vaccinated after symptom onset were included and classified as unvaccinated.

Of the included 10,484 outpatients, 3,409 (32.5%) tested positive and 7,075 (67.5%) were negative. Among the positive results, 3,382 were for influenza A(H3N2), nine were for influenza A(H1N1)pdm09, and 18 for influenza B viruses. Positivity rate peaked in week 49 at 62.7%, lower than the influenza A(H3N2)-dominated season in spring 2023 [12].

The median age was 25 years (interquartile range (IQR): 13.0–39.0) for cases and 29 years (IQR: 14.5–41.0) for controls. School-aged children (6–17 years) accounted for a larger proportion of cases (33.2% vs 22.7% controls), and comorbidities were less common among cases (5.5% vs 7.2% controls) (Table 1). We provided detailed information on the vaccination status of cases and controls in Supplementary Table S1.

**Table 1:** Descriptive characteristics of enrolled patients with influenza-like illness, Beijing, China, weeks 40/2025–04/2026 2025 (n = 10,484)

Characteristics	Overall (n =10,484)		Influenza-positive cases (n = 3,409)		Test-negative controls (n = 7,075)		p value^a^
	n	%	n	%	n	%	
Median age in years (IQR)	27.0 (14.0–41.0)		25.0 (13.0–39.0)		29.0 (14.5–41.0)		< 0.001
Age group							
≤ 5 years	909	8.7	208	6.1	701	9.9	< 0.001
6–17 years	2,737	26.1	1,133	33.2	1,604	22.7	
18–59 years	5,569	53.1	1,697	49.8	3,872	54.7	
≥ 60 years	1,269	12.1	371	10.9	898	12.7	
Sex							
Female	5,422	51.7	1,731	50.8	3,691	52.2	0.188
Male	5,062	48.3	1,678	49.2	3,384	47.8	
Resident area^b^							
Urban	4,468	42.6	1,479	43.4	2,989	42.2	0.449
Suburban	5,756	54.9	1,842	54.0	3,914	55.3	
Unknown	260	2.5	88	2.6	172	2.4	
Comorbidity^c^							
No	9,784	93.3	3,221	94.5	6,563	92.8	0.001
Yes	700	6.7	188	5.5	512	7.2	
Vaccination status							
Non-vaccinated	9,021	86.0	2,835	83.2	6,186	87.4	<0.001
Vaccinated^d^	1,463	14.0	574	16.8	889	12.6	

ILI: influenza-like illness; IQR: interquartile range.

^a^ The p value for median age in years was calculated using the Wilcoxon test, while p values for other factors are calculated using the chi-square test. A p value of 0.05 was considered significant.

^b^ Resident area is classified as urban, suburban or unknown. The urban area includes six core urban districts and the Beijing Economic-Technological Development Area; the remaining 10 districts were classified as suburban. ‘Unknown’ indicates missing information on area of residence.

^c^ Comorbidities include chronic obstructive pulmonary disease, asthma, cardiovascular disease, diabetes, immunodeficiency or organ transplant, renal impairment, rheumatologic disease, neuromuscular disease, cirrhosis or liver disease, neoplasms, autoimmune diseases or haematological diseases.

^d^ Among vaccinated participants, the majority (99.9%) received inactivated influenza vaccines, including trivalent split-virus formulations (85.7%), quadrivalent split-virus formulations (11.4%), and quadrivalent subunit formulations (2.8%). Only 0.1% received live attenuated influenza vaccine.

Influenza-positive cases were defined as ILI patients with PCR-confirmed influenza; test-negative controls were ILI patients who tested negative.

## Phylogenetic analysis and antigenic characterisation

Whole genome sequencing of 316 randomly selected influenza A(H3N2)-positive specimens showed predominance of subclade K (268/316; 84.8%), followed by J.2.2 (36/316; 11.4%), J.2.4 (7/316; 2.2%) and J.2 (5/316; 1.6%). Compared with the 2025/26 northern hemisphere vaccine strain (A/Croatia/10136RV/2023 (H3N2)-like), subclade K viruses carried multiple HA amino acid substitutions, including K2N, T135K, S144N, N145S, N158D, I160K, Q173R, A186D, K189R, T328A and S378N. We provide a phylogenetic tree of 11 randomly selected sequences from different clades in Supplementary Figure S3.

Of 67 virus isolates recovered for antigenicity analyses, 65 showed reduced reactivity to the vaccine strain, whereas two remained antigenically similar.

## Vaccine effectiveness estimates

Vaccination rate varied substantially across age groups (Table 2). Children aged 6–17 years had the highest rate in both influenza-positive cases and test-negative controls (40.2% and 38.2%, respectively). Vaccination rate was substantially lower among adults aged 18–59 years (2.4% in cases and 2.6% in controls). In children aged ≤ 5 years, vaccination rate was lower among controls than cases (8.0% vs 10.1%), and a similar pattern was observed among adults aged ≥ 60 years (13.1% vs 15.6%). Vaccination rates by age and calendar week strata are shown in Supplementary Figures S1 and S2.

**Table 2:** Influenza vaccine effectiveness estimated using a test-negative design, Beijing, China, weeks 40/2025–04/2026 2025 (n = 10,484)

Characteristics	Total	Influenza-positive cases			Test-negative controls			Crude VE		Adjusted VE^a^	
		Vaccinated	Total	%	Vaccinated	Total	%	%	95% CI	%	95% CI
Overall	10,484	574	3,409	16.8	889	7,075	12.6	−40.9	−57.9 to −25.7	23.5	11.7 to 33.7
A(H3N2)	10,457	573	3,382	16.9	889	7,075	12.6	−41.9	−59.1 to −26.6	23.3	11.5 to 33.5
Age groups											
≤ 5 years	909	21	208	10.1	56	701	8.0	−29.3	−119.2 to 23.7	19.6	−44.3 to 55.2
6–17 years	2,737	455	1,133	40.2	613	1,604	38.2	−8.5	−26.8 to 7.2	19.5	3.3 to 33.0
18–59 years	5,569	40	1,697	2.4	102	3,872	2.6	10.8	−29.2 to 38.4	41.1	12.7 to 60.2
≥ 60 years	1,269	58	371	15.6	118	898	13.1	−22.5	−72.2 to 12.9	13.4	−27.5 to 41.2

CI: confidence interval; VE: vaccine effectiveness.

^a^ The overall VE against influenza is obtained by adjusting for age (modelled as a natural cubic spline), sex, resident area, calendar week (modelled as a natural cubic spline) and comorbidities. The VE by age group is adjusted for sex, resident area, calendar week (modelled as a natural cubic spline), comorbidities and age (modelled as a natural cubic spline). The VE against influenza subtype H3N2 is adjusted for age (modelled as a natural cubic spline), sex, resident area, calendar week (modelled as a natural cubic spline) and comorbidities.

After adjustment for age, sex, comorbidities, resident area and calendar week, the overall VE against laboratory-confirmed influenza was 23.5% (95% CI: 11.7–33.7). Specifically, the adjusted VE was 23.3% (95% CI: 11.5–33.5) for influenza A(H3N2). In age-stratified analyses, the overall adjusted VE was 19.6% (95% CI: −44.3 to 55.2) among children ≤ 5 years, 19.5% (95% CI: 3.3–33.0) for 6–17 years, 41.1% (95% CI: 12.7–60.2) for 18–59 years, and 13.4% (95% CI:  −27.5 to 41.2) for ≥ 60 years (Table 2). In Supplementary Table S2, we append sensitivity analyses, in which different vaccination definitions and enrolment periods yielded consistent results.

## Discussion

In this early 2025/26 influenza season in Beijing, China, influenza A(H3N2) subclade K viruses predominated. Despite prior studies suggesting that post-infection ferret antisera and human post-vaccination sera poorly recognise subclade K virus [2,6], we observed a modest real-world VE in this season (23.5%; 95% CI: 11.7–33.7), comparable with VE observed in previous A(H3N2) seasons in Beijing (9.5% in 2022/23, 37.6% in 2023/24) [13,14].

Although crude VE estimates were negative, the adjusted VE was 23.5% after accounting for age, calendar week and other covariates. This apparent reversal is attributable to strong confounding by age and calendar week, reflecting differential vaccine uptake and influenza risk across the season. Within each age–calendar week stratum, vaccination rate was generally lower among influenza-positive cases than among influenza-negative controls, indicating a protective effect of vaccination at the stratum level. Adjustment for calendar week and age therefore mitigated this confounding, resulting in a more accurate estimate of vaccine effectiveness.

Our VE estimates for working-age adults (41.1%) were comparable with those reported from England (59.9%), France (45.1%) and the European Centre for Disease Prevention and Control (ECDC) (57.0%), whereas VE estimates for children and older adults were relatively lower, which may reflect differences in vaccine formulations and case definitions [6-8]. Among vaccinated participants in our study, the vast majority (99.9%) received inactivated influenza vaccines. Among children, the VE was 74.7% in England, where live attenuated influenza vaccine is primarily used, whereas the VE for children our study was 19.5%, within the confidence interval reported from Canada (36%; 10–55%) but lower than estimates from France (57.2%) and the ECDC (52.0%) [6-8,15]. Among older adults (≥ 65 years), VE was 34.8% in England and 27.7% in France, where enhanced vaccines in high-dose and adjuvanted formulations are preferentially recommended for this population [6,8]. Randomised controlled trials and case–control studies have shown that enhanced formulations could improve immune responses and protection in this age group [16-19]. Differences in VE across regions may also reflect variations in case definitions. The study in England included patients presenting to emergency departments, France enrolled outpatients, the ECDC included acute respiratory infection (ARI) or ILI, and Canada enrolled ARI patients, which may represent different disease severities [6-8,15].

Nevertheless, the overall evidence indicates that, despite antigenic drift and mismatch between the vaccine strain and circulating viruses, influenza vaccination remained effective, which supports its continued promotion. Although the estimated VE of 23.5% represents modest individual protection, it is still epidemiologically meaningful, reducing influenza cases and limiting onward transmission at the population level.

This study has some limitations. Firstly, comorbidity status was self-reported, which may have led to misclassification. Secondly, although virological surveillance demonstrated predominance of influenza A(H3N2) subclade K, the sample size was insufficient to estimate vaccine effectiveness specifically against subclade K infections. Finally, we applied a 3-day window, which may have captured a greater proportion of patients in the acute phase of illness, potentially reflecting differences in healthcare-seeking behaviour compared with a 7-day window. However, as the same inclusion criteria were applied equally to both influenza test-positive cases and test-negative controls, the impact on VE estimates is likely to be limited. 

## Conclusions

Our study demonstrates that influenza vaccination provided overall modest protection against A(H3N2) subclade K viruses in Beijing, China, despite the substantial genetic and antigenic differences from the vaccine strain. These findings support the ongoing public health value of influenza vaccination and highlight the importance of continuous virological surveillance and timely evaluation of VE against emerging influenza subclades.

## Data Availability

Sequencing data of influenza virus strains are deposited in the Global Initiative for Sharing All Influenza Data (GISAID) under accession numbers: EPI_ISL_20313621 to EPI_ISL_20313743, EPI_ISL_20313746 to EPI_ISL_20313925, EPI_ISL_20313927 to EPI_ISL_20313930, EPI_ISL_20313932 to EPI_ISL_20313934, EPI_ISL_20313936 to EPI_ISL_20313937, and EPI_ISL_20313989 to EPI_ISL_20313992, 316 sequences in total.

## Supplementary Data

1066000 Supplement
