# Supplementary material for: Modest protection from vaccination against influenza A(H3N2) subclade K, Beijing, China, 2025/26 season
Source: Euro Surveill. 2026 Feb 19;31(7):2600096. doi: 10.2807/1560-7917.ES.2026.31.7.2600096 (PMC12923999; doi:10.2807/1560-7917.ES.2026.31.7.2600096)
Supplement: Supplement [file 26-00096_YANG_Supplement.pdf]

Supplementary materials

This supplementary material is hosted by Eurosurveillance as supporting information alongside the article "Modest protection from vaccination against influenza A(H3N2) subclade K, Beijing, China, 2025/26 season", on behalf of the authors, who remain responsible for the accuracy and appropriateness of the content. The same standards for ethics, copyright, attributions and permissions as for the article apply. Supplements are not edited by Eurosurveillance and the journal is not responsible for the maintenance of any links or email addresses provided therein.

Figure S1. Weekly enrolment and vaccination rate among influenza-positive cases and test-negative controls, Beijing, China, weeks 36/2025 to 04/2026

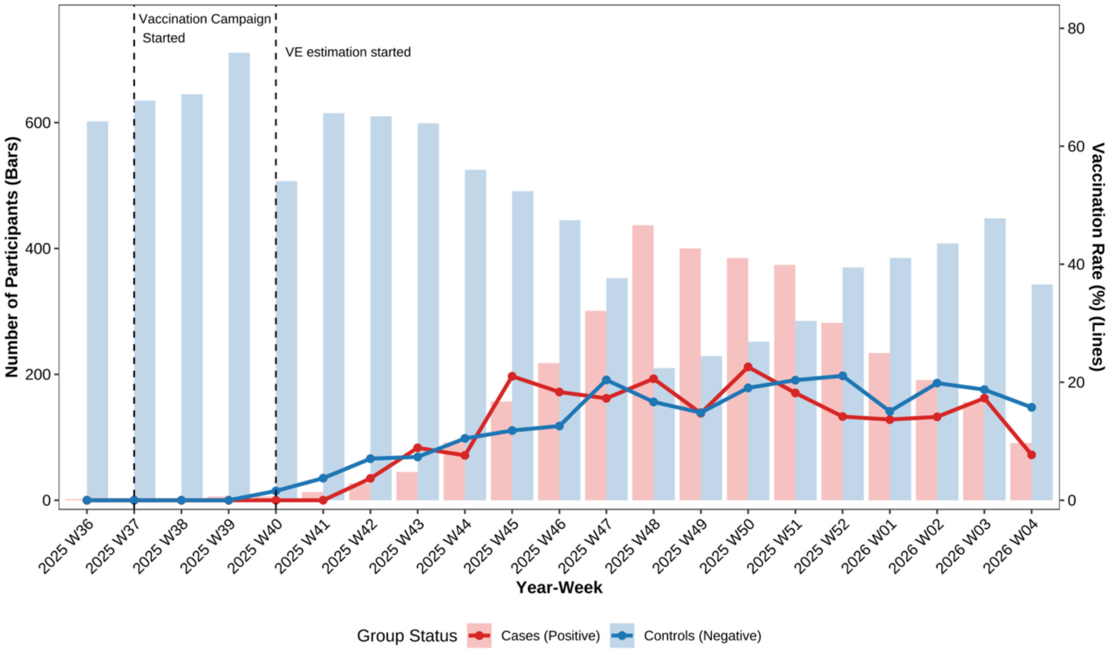

Note: The left vertical axis (y-axis) and the bars represent the total number of participants enrolled each week. The right vertical axis and the connected lines indicate the weekly influenza vaccination rate (%). Red bars and lines represent influenza-positive cases, while blue bars and lines represent test-negative controls. Timeframe shown by year and week (ISO week of sample date), starting from week 36/2025. Vaccination rate for each week is calculated as the proportion of vaccinated individuals among the total number of cases or controls enrolled in that specific week.

The first vertical dashed line marks the start of Beijing's 2025/26 mass influenza vaccination campaign (week 37/2025). The second vertical dashed line indicates the start of vaccine effectiveness (VE) estimation (week 40/2025), which is the first full week after two weeks following the vaccination campaign.

In Beijing, the 2025/26 mass influenza vaccination campaign followed a multi-tiered approach. Free vaccination was provided to primary and secondary school students via school-based programs, to permanent local residents aged≥ 60 years through community-led outreach, and to healthcare workers, school teachers, staff in long-term care facilities, etc. Other residents had access to vaccination through a self-paid, voluntary scheme. Therefore, vaccination coverage was the highest among school-aged children and the elderly.

Figure S2. Weekly enrolment and vaccination rate among influenza-positive cases and test-negative controls by age group, Beijing, China, weeks 36/2025 to 04/2026

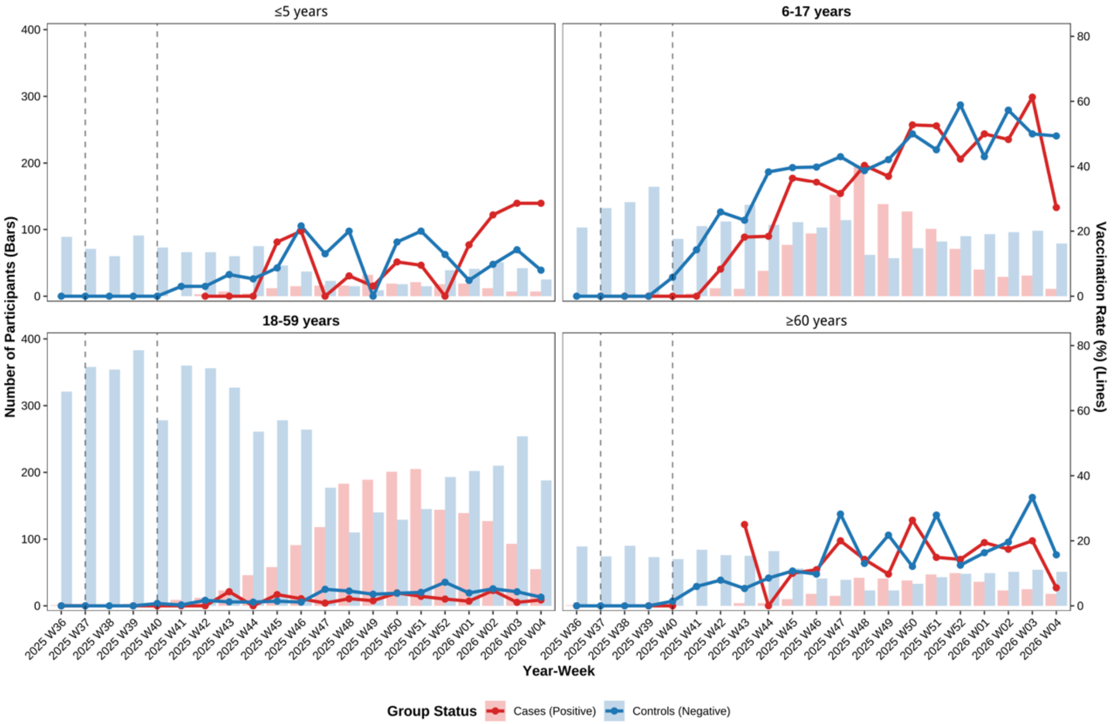

Note: The left vertical axis (y-axis) and the bars represent the total number of participants enrolled each week. The right vertical axis and the connected lines indicate the weekly influenza vaccination rate (%). Red bars and lines represent influenza-positive cases, while blue bars and lines represent test-negative controls. Data are presented in four age-specific panels: ≤5 years, 6–17 years, 18–59 years, and ≥60 years.

The first vertical dashed line marks the start of Beijing's 2025/26 mass influenza vaccination campaign (week 37/2025). The second vertical dashed line indicates the start of vaccine effectiveness (VE) estimation (week 40/2025), which is the first full week after two weeks following the vaccination campaign. Vaccination rate for each week is calculated as the proportion of vaccinated individuals among the total number of cases or controls enrolled in that specific week. In weeks where the number of cases or controls is zero, the corresponding bars and data points are omitted.

Table S1. Vaccination status of influenza-positive cases and test-negative controls by participant characteristics, Beijing, China, weeks 40/2025 to 04/2026 (n =10,484)

Characteristics	Total	Influenza-positive cases			Test-negative controls		
		Vaccinated	Total	% [a]	Vaccinated	Total	%
Age groups							
≤ 5 years	909	21	208	10.1	56	701	8.0
6–17 years	2,737	455	1,133	40.2	613	1,604	38.2
18–59 years	5,569	40	1,697	2.4	102	3,872	2.6
≥ 60 years	1,269	58	371	15.6	118	898	13.1
Sex							
Female	5,422	262	1,731	15.1	432	3,691	11.7
Male	5,062	312	1,678	18.6	457	3,384	13.5
Resident area[b]							
Urban	4,468	254	1,479	17.2	383	2,989	12.8
Suburban	5,756	317	1,842	17.2	494	3,914	12.6
Unknown	260	3	88	3.4	12	172	7.0
Comorbidities[c]							
No	9,784	546	3,221	17.0	832	6,563	12.7
Yes	700	28	188	14.9	57	512	11.1
Time since vaccination to disease onset[d]							
14 -30 d	243	57		9.9	186		20.9
31-60 d	506	219		38.2	287		32.3
60-90 d	475	235		40.9	240		27.0
≥90 d	239	63		11.0	176		19.8

Note:

[a] For age groups, sex, resident area and comorbidities, percentages represent the vaccination rate within each category. For “Time since vaccination to disease onset,” percentages indicate the proportion of vaccinated participants in each time interval among all vaccinated individuals in cases or controls.

[b] Resident area is classified as urban, suburban or unknown. Beijing comprises 17 administrative districts. The urban area includes six core urban districts (Dongcheng, Xicheng, Chaoyang, Haidian, Fengtai, and Shijingshan) as well as the Beijing Economic-Technological Development Area; all remaining districts were classified as suburban. “Unknown” indicated missing information on area of residence.

[c] Comorbidities include chronic obstructive pulmonary disease, asthma, cardiovascular disease, diabetes, immunodeficiency or organ transplant, renal impairment, rheumatologic disease, neuromuscular disease, cirrhosis or liver disease, neoplasms, autoimmune diseases or haematological diseases.

[d] Vaccination was defined as receipt of influenza vaccine at least 14 days before symptom onset. Post-onset vaccination was classified as unvaccinated. Time since vaccination to disease onset was calculated only among vaccinated participants with an interval of ≥14 days between vaccination and symptom onset.

Influenza-positive cases were defined as ILI patients with PCR-confirmed influenza; test-negative controls were ILI patients who tested negative.

Figure S3. Phylogenetic analysis of the haemagglutinin (HA) gene of the influenza H3N2 compared to reference vaccine strains, Beijing, China

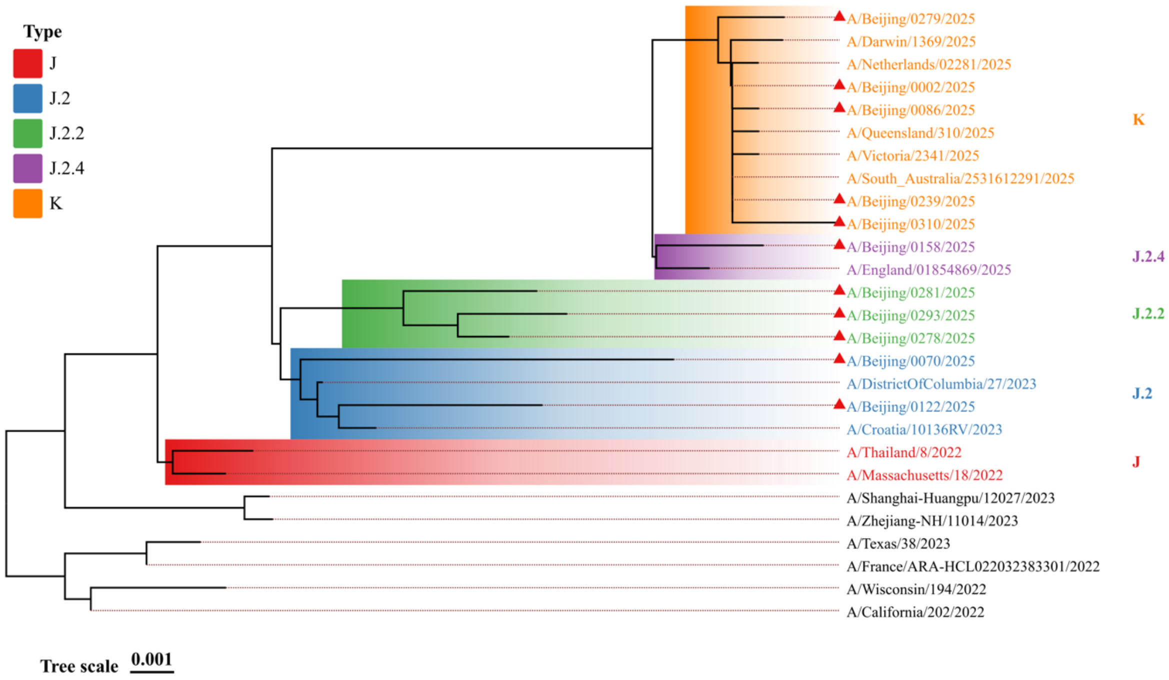

Table S2. Sensitivity analyses of influenza vaccine effectiveness under alternative vaccination classifications and enrollment period, Beijing, China, 2025/26 season

Characteristics	Total	Influenza-positive cases			Test-negative controls			Crude VE		Adjusted VE[a]	
		Vaccinated	Total	%	Vaccinated	Total	%	%	95% CI	%	95% CI
Primary analysis	10,484	574	3,409	16.8	889	7,075	12.6	-40.9	-57.9 to -25.7	23.5	11.7 to 33.7
Sensitivity analysis											
Scenario 1	10,213	574	3,392	16.9	889	6,821	13.0	-35.9	-52.3 to -21.3	22.9	11.1 to 33.1
Scenario 2	10,625	574	3,437	16.7	889	7,188	12.4	-42.1	-59.2 to -26.8	23.5	11.8 to 33.7
Scenario 3	10,354	574	3,420	16.8	889	6,934	12.8	-37.1	-53.7 to -22.4	23.4	11.7 to 33.5
Scenario 4	8,706	573	3,363	17.0	815	5,343	15.3	-14.1	-28.2 to -1.5	22.3	10.3 to 32.7
Scenario 5	8,062	569	3,318	17.1	771	4,744	16.3	-6.7	-20.1 to 5.3	22.8	10.8 to 33.3

Note:

Primary analysis: participants vaccinated after symptom onset (post-onset vaccinated, n=271) were classified as unvaccinated and 0-14 days before symptom onset (0-14 days pre-onset, n=141) were excluded; enrollment started 2 weeks after mass vaccination (weeks 40/2025–04/2026).

Among the 271 post-onset vaccinated individuals, 42 were those aged ≤5 years, 88 were 6–17 years, 73 were 18–59 years, and 68 were ≥60 years. Among the 141 individuals vaccinated 0–14 days pre-onset, 16 were ≤5 years, 88 were 6–17 years, 19 were 18–59 years, and 18 were ≥60 years.

Vaccination classification sensitivity analyses to test how vaccination definitions may affect VE estimation:

Scenario 1: post-onset and 0-14 days pre-onset vaccinated both excluded.

Scenario 2: post-onset and 0-14 days pre-onset vaccinated both classified as unvaccinated.

Scenario 3: post-onset vaccinated excluded; 0-14 days pre-onset classified as unvaccinated. (Enrollment period: weeks 40/2025–04/2026 for Scenarios 1–3.)

Enrollment period sensitivity analyses: to test the robustness of VE estimates to different start weeks.

Scenario 4: enrollment started at first week with influenza positivity ≥5% (weeks 43/2025–04/2026).

Scenario 5: enrollment started at first week with influenza positivity ≥10% (weeks 44/2025–04/2026).

VE was re-estimated under each scenario, adjusting for age (modeled as a natural cubic spline), sex, resident area, calendar week (modeled as a natural cubic spline) and comorbidities.
